# Immunoassay for Capsular Antigen of *Bacillus anthracis* Enables Rapid Diagnosis in a Rabbit Model of Inhalational Anthrax

**DOI:** 10.1371/journal.pone.0126304

**Published:** 2015-05-05

**Authors:** Marcellene A. Gates-Hollingsworth, Mark R. Perry, Hongjing Chen, James Needham, Raymond L. Houghton, Syamal Raychaudhuri, Mark A. Hubbard, Thomas R. Kozel

**Affiliations:** 1 University of Nevada School of Medicine, Reno, Nevada, United States of America; 2 Battelle Biomedical Research Center, Columbus, Ohio, United States of America; 3 InBios International, Inc., Seattle, Washington, United States of America; George Mason University, UNITED STATES

## Abstract

Inhalational anthrax is a serious biothreat. Effective antibiotic treatment of inhalational anthrax requires early diagnosis; the further the disease has progressed, the less the likelihood for cure. Current means for diagnosis such as blood culture require several days to a result and require advanced laboratory infrastructure. An alternative approach to diagnosis is detection of a *Bacillus anthracis* antigen that is shed into blood and can be detected by rapid immunoassay. The goal of the study was to evaluate detection of poly-γ-D-glutamic acid (PGA), the capsular antigen of *B*. *anthracis*, as a biomarker surrogate for blood culture in a rabbit model of inhalational anthrax. The mean time to a positive blood culture was 26 ± 5.7 h (mean ± standard deviation), whereas the mean time to a positive ELISA was 22 ± 4.2 h; P = 0.005 in comparison with blood culture. A lateral flow immunoassay was constructed for detection of PGA in plasma at concentrations of less than 1 ng PGA/ml. Use of the lateral flow immunoassay for detection of PGA in the rabbit model found that antigen was detected somewhat earlier than the earliest time point at which the blood culture became positive. The low cost, ease of use, and rapid time to result of the lateral flow immunoassay format make an immunoassay for PGA a viable surrogate for blood culture for detection of infection in individuals who have a likelihood of exposure to *B*. *anthracis*.

## Introduction

*Bacillus anthracis*, the causative agent of inhalational anthrax, is a major biothreat. Lucey has proposed a four-stage classification for the progression of inhalational anthrax: asymptomatic, early-prodromal, intermediate-progressive and late-fulminant [1]. The efficacy for treatment of inhalational anthrax depends on the stage at which antibiotics are administered. The further the disease has progressed, the less the likelihood for cure. Few patients will survive if untreated disease progresses to the late-fulminant stage.

Despite the critical role for early diagnosis in treatment and patient outcome, there has been little change in the approach to laboratory diagnostic methods for inhalational anthrax; blood culture remains the gold standard for diagnosis of anthrax. During the 2001 anthrax attacks, reference laboratories were overwhelmed with samples that required a minimum of 24 h for a negative culture and between 3–4 days to confirm positive cases [2]. The critical timeframe for initiating successful treatment, combined with the incubation period required for blood culture, underscores the need for a more rapid and sensitive diagnostic test for inhalational anthrax. Additionally, the potential for a large-scale bioterrorism event emphasizes the need for a test that can be performed quickly, easily and effectively on a large population of patients, free of the constraints of current tests that require advanced laboratory infrastructure and highly trained personnel.

*B*. *anthracis* is surrounded by a polypeptide capsule composed of gamma-linked poly-d-glutamic acid (PGA). Our laboratory previously created a library of monoclonal antibodies (mAbs) that react with this capsular antigen [3]. An immunoassay for PGA constructed from capsular mAbs enabled us to determine that circulating PGA is shed into blood in mouse and non-human primate models of inhalational anthrax [3–5].

Past studies have demonstrated that the pathology of inhalational anthrax in non-human primates and rabbits closely resembles that observed in humans following aerosol exposure to *B*. *anthracis* [6–8]. In this study, we used a rabbit model to i) assay plasma and urine PGA levels during the course of inhalational anthrax to identify the immunoassay sensitivity needed for early diagnosis of infection, ii) construct a rapid anthrax immunoassay for PGA that provides the sensitivity needed for early diagnosis, and iii) evaluate the performance of a rapid immunoassay for early diagnosis of inhalational anthrax.

## Materials and Methods

### Bacillus spp. and isolation of PGA

*B*. *anthracis* Ames strain spores were produced and maintained at Battelle Biomedical Research Center (Columbus, OH). *B*. *anthracis* Pasteur is maintained by the Nevada State Public Health Laboratory (Reno, NV) and was originally obtained from the Centers for Disease Control and Prevention. *Bacillus licheniformis* strain 9945 was obtained from the American Type Culture Collection (Manassas, VA). PGA was isolated from *B*. *anthracis* Pasteur and *Bacillus licheniformis* cultures as described [3,9].

### mAb production

The murine IgG3 mAb F26G3 was produced from BALB/c mice that were immunized with *B*. *licheniformis* PGA in combination with CD40 agonist antibodies [3,9,10]. mAb 8B10 was produced by subcutaneous immunization of CD1 mice with a synthetic γDPGA oligomer coupled to KLH and emulsified with TiterMax Gold (Titermax, Norcross, GA). Six weeks later, mice were given an intravenous booster with *B*. *licheniformis* PGA, and spleens were harvested 3 days later for the production of hybridomas. Hybridomas were produced by fusion of splenocytes with the p3X63Ag8.653 cell line (Sigma) using standard techniques. PGA mAbs were evaluated using an enzyme-linked immunosorbent assay (ELISA) as described [3].

### mAb Affinity

Surface plasmon resonance was used to assess the functional affinities of the two mAbs, F26G3 and 8B10. Using a Biacore X100 instrument, 100 RU (response units) of a 25-residue synthetic γDPGA oligomer were immobilized on the surface of a CM5 sensor chip (GE Healthcare). Antibody binding was measured by injecting each mAb at multiple concentrations over the immobilized PGA sensor surface [10]. The dissociation constant (K_D_) was calculated using the steady-state equilibrium model in BIAevaluation software.

### Rabbit model of inhalational anthrax

Ten (5 male and 5 female) New Zealand white rabbits were purchased from Covance Laboratories (Denver, PA) and placed into quarantine upon arrival at Battelle Biomedical Research Center. *B*. *anthracis* Ames spores were prepared and characterized as previously described [11,12] and were used to prepare nebulization suspensions of *B*. *anthracis* spores in 0.01 percent Tween 80 at a concentration of approximately 1.46 x 10^9^ colony forming units (CFU)/ml. The target inhaled dose was 200 median lethal doses (LD_50_; 105,000 CFU/animal established by Zaucha et al., 1998) [8]. The actual inhaled dose was calculated from the *B*. *anthracis* spore aerosol concentration and the total accumulated tidal volume. The animals received an average inhaled dose of 340 ± 55 LD_50_.

Blood and urine were collected and body temperature was determined pre-exposure and 12, 18, 24, 30, 36 and 48 h post challenge. The exception was the absence of some early urine samples due to complications from anesthesia. Briefly, animals were initially sedated using a xylazine/ketamine sedative for collecting blood (ear artery or vein) and urine (cystocentesis). Administration of the sedative resulted in micturition before cystocentesis could be performed. The protocol was amended after the 24 h time-point to collect urine in a clean waste tray that was placed in each cage 2 h prior and remained for up to 4 h (± 2 h of the targeted time-point), and xylazine/ketamine was replaced with acepromazine for blood collection. A portion of each whole blood sample, taken with a sterile loop (30 μl–40 μl), was tested for bacteremia. Blood samples were streaked over blood agar plates and read for growth and morphology consistent with *B*. *anthracis* after a minimum incubation of 48 hours at 37°C. Plasma samples were filter sterilized and stored at ≤ -70°C. Body temperature was monitored by a transponder chip implanted at the shoulder blade and febricity was determined by comparison to a pre-exposure baseline established for each rabbit.

### Quantitative antigen-capture ELISA for PGA

Microtiter plates were coated overnight with mAb F26G3 (1 μg/ml) in PBS, washed with PBS-Tween (PBS containing 0.05% Tween 20), and blocked for 90 min with PBS-Tween. Samples of plasma or urine were serially diluted in PBS-Tween and incubated for 90 min with the mAb-coated wells. Plates were washed with PBS-Tween, incubated for 90 min with horseradish peroxidase-labeled mAb F26G3 (1 μg/ml) diluted in PBS-Tween with 0.05% milk, washed and then incubated with tetramethylbenzidine substrate (Kirkegaard & Perry Laboratories, Inc., Gaithersburg, MD). The reaction was stopped after 30 min with a solution of 1 M H_3_PO_4_ and plates were read at an optical density of 450 nm (OD_450_). Purified *B*. *anthracis* Pasteur PGA diluted in PBS-Tween was used as a standard for calculating PGA levels in rabbit plasma or urine. The concentration of purified PGA standard that produced an OD of 0.5 in a log-log plot of OD_450_ vs. ng PGA/ml was calculated as the endpoint and used to determine PGA levels in the plasma and urine. The lower limit of detection of the PGA ELISA, calculated as a 3-fold increase over background, was 40 pg/ml.

### Lateral flow immunoassay (LFA) for PGA

mAb 8B10 was passively absorbed to 40 nm colloidal gold particles at 1.5 μg/ml, concentrated to OD 6 and sprayed onto a conjugate pad at 6 μl/cm using a BioDot XYZ3000. For the test line, 0.20 mg/ml 8B10 was sprayed at 1 μl/cm onto nitrocellulose membrane. An additional control line reactive with gold conjugate was included to verify proper flow between membranes. Assays were housed in a plastic cassette for ease of use. Cassettes provided a port for sample application and test and control lines were framed by a viewing window.

The limit of detection (LOD) of the LFA constructed with mAb 8B10 was determined using purified *B*. *anthracis* Pasteur PGA diluted in pooled normal rabbit plasma and normal human serum. Twenty microliters were applied into the sample port and then followed with 2 drops of a chase buffer. After 15 min, blinded assays were read by four different individuals. Plasma samples from infected rabbits were evaluated for presence of PGA in a similar fashion. Results were reported as positive if at least 3 out of 4 evaluators deemed the assay to be positive.

### Statistics

Affinity results from repeated experiments are reported as the mean and standard error; comparison was done by a Student’s *t*-test. The time to a positive result with LFA and ELISA were compared to the time to a positive result by blood culture using the One Way Repeated Measures Analysis of Variance. Post hoc evaluation of time to each immunoassay result vs. time to positive blood culture was done with the Bonferroni *t* test. A log transformation was used to assess the geometric mean of the PGA concentration at the time of the first positive blood culture. All calculations were done using the statistical analysis component of SigmaPlot (San Jose, CA).

### Ethics Statement

This study was approved by the University of Nevada, Reno (Permit Number: 00024) and Battelle Biomedical Research Center (Permit Number: 2460) Institutional Animal Care and Use Committees and followed the principles of the Guide for the Care and Use of Laboratory Animals of the National Research Council. Animals were anesthetized with xylazine/ketamine or acepromazine for sample collection and surviving animals were euthanized at the end of the study using an American Veterinary Medical Association (AVMA) accepted method of euthanasia.

## Results and Discussion

**Fig 1:**
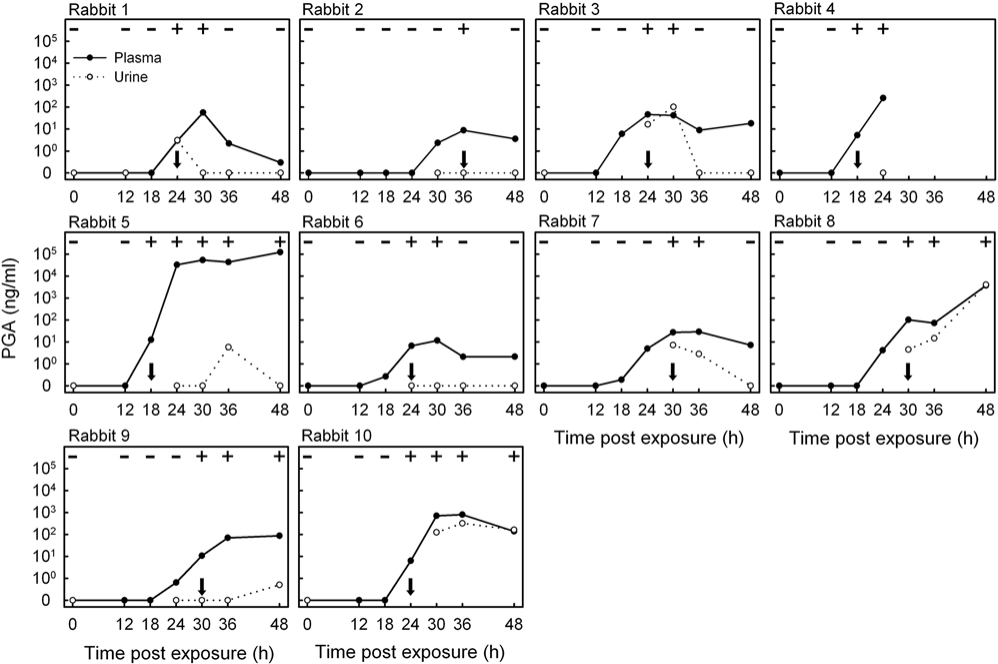
ELISA analysis of the concentration of poly-γ-D-glutamic acid (PGA) in plasma and urine collected in a time-point series from ten rabbits after inhalational exposure to *B*. *anthracis* spores. Parallel blood culture results are reported at the top of each graph as negative (-) or positive (+); arrows identify the earliest point infection was detected by blood culture.

An ELISA was constructed with the IgG3 anti-PGA mAb F26G3 and was used to quantify PGA levels in rabbit plasma and urine collected in a time-point series following inhalational exposure to *B*. *anthracis* Ames spores. The level of PGA in plasma and urine was plotted for the time course of infection for each rabbit and compared with blood culture results (Fig 1). Detectable PGA concentrations in plasma ranged over the course of infection from a low of 0.2 ng PGA/ml (Rabbit 7, 18 h) to a high of 120,000 ng/ml (Rabbit 5, 48 h) and ranged from 2.9 to 100 ng PGA/ml at the earliest time when parallel blood cultures retrospectively became positive (Table 1). The geometric mean of the PGA concentration at the time of the first positive blood culture was 13 ng PGA/ml (99% confidence interval on the geometric mean = 5.2–30 ng PGA/ml). In all ten rabbits, the quantitative assay detected PGA in plasma at the time of the first positive blood culture and frequently identified the infection sooner, when cultures were still negative. The mean time to a positive blood culture was 26 ± 5.7 h (mean ± standard deviation). The mean time to a positive ELISA was 22 ± 4.2 h (mean ± standard deviation; *P* = 0.005 by One Way Repeated Measures ANOVA with post hoc evaluation by Bonferroni *t*-test). Fever (temperature rise of ≥2°F) occurred slightly later than time of the first positive blood culture (median = 28 ± 5.7 h).

**Table 1:** Comparison of sensitivity of lateral flow immunoassay (LFA), enzyme-linked immunosorbent assay (ELISA), blood culture and temperature rise for diagnosis of inhalational anthrax.

Rabbit	Time to positive result (h post challenge)				PGA^a^ plasma concentration (ng/ml) at time of first positive result	
	LFA^b^ (plasma)	ELISA^c^ (plasma)	Blood culture	Temperature rise of ≥2°F	Blood culture^d^	Temperature rise of ≥2°F
1	24	24	24	36	2.9	2.2
2	30	30	36	36	8.7	8.7
3	18	18	24	18	46	6.0
4	18	18	18	24	5.2	260
5	18	18	18	24	13	33,000
6	12	18	24	30	6.6	12
7	18	18	30	24	27	4.9
8	24	24	30	30	100	100
9	24	24	30	30	11	11
10	24	24	24	30	6.3	700

^a^Poly-γ-D-glutamic acid (PGA)

^b^LFA time to positive vs. blood culture time to positive: *P* = 0.002 by One Way Repeated Measures ANOVA with post hoc evaluation by Bonferroni *t*-test

^c^ELISA time to positive vs. blood culture time to positive: *P* = 0.005 by One Way Repeated Measures ANOVA with post hoc evaluation by Bonferroni *t*-test

^d^Descriptive statistics: median = 9.8 ng/ml, lower quartile = 6.3, upper quartile = 27; geometric mean = 13 ng/ml with a 99% confidence interval of 5.2–30

Although PGA was readily detectable in plasma early in the course of infection, urine proved to be a less reliable sample for detection of antigen (Fig 1). PGA was found in urine in only 7 of 10 animals and overall was not a consistent early indicator of disease. Appearance of PGA in urine lagged behind both the development of bacteremia as shown by positive blood culture and the appearance of PGA in plasma.

The LFA is a membrane-based assay that can be used for the detection of antigen or antibody and has become an increasingly popular format for rapid diagnostics. Although design and optimization of the assay can often be complex, performance of the assay itself is very simple, providing a positive or negative result within 15 minutes that can be read visually without the need for instrumentation. As a consequence, the LFA platform was chosen for construction of a rapid diagnostic test. A target sensitivity of ≤ 2 ng PGA/ml plasma was set for LFA construction based on results of the quantitative evaluation of PGA in plasma at the time of the first positive blood culture (geometric mean – 13 ng/ml with a 99% confidence interval of 5.2–30 ng PGA/ml plasma; Fig 1 and Table 1).

mAb F26G3 was used for construction of the initial ELISA that determined concentrations of PGA in plasma from infected animals (Fig 1). However, mAb F26G3 is a murine IgG3 that self-associated when used in the LFA format and produced a demonstrable non-specific background reaction. As a consequence, a new set of PGA immunizations was performed, and new hybridomas that produced non-IgG3 mAbs were selected. An IgG1 mAb, designated 8B10, was chosen for LFA development based on high sensitivity for detection of PGA in the sandwich ELISA format and surface plasmon resonance results showing mAb 8B10, K_D_ = 5.2 nM ± 1.5, to have a significantly higher affinity (*P* = 0.029) compared to mAb F26G3, K_D_ = 10 nM ± 1.1.

**Fig 2:**
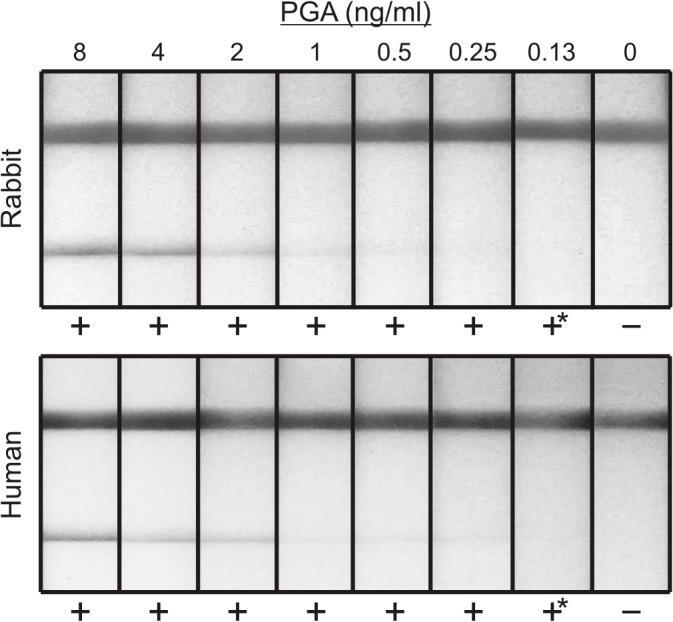
Sensitivity of LFA for detection of purified poly-γ-D-glutamic acid (PGA) in pooled normal rabbit plasma and human serum. The top line is a control indicating proper flow of the lateral flow device. The bottom line is the test line for detection of PGA. Results of visual evaluation by four individuals are reported as negative (-) or positive (+) below each assay; (*) denotes a result that was considered weak, but still deemed positive by at least 3 out of 4 evaluators.

An anthrax LFA was constructed with mAb 8B10, and the sensitivity was assessed using purified PGA spiked into pooled normal rabbit plasma and human serum (Fig 2). The LOD of the rapid anthrax diagnostic in both matrices was 0.13 ng PGA/ml, demonstrating a >10-fold higher sensitivity than the sensitivity goal set for early diagnosis of infection.

**Fig 3:**
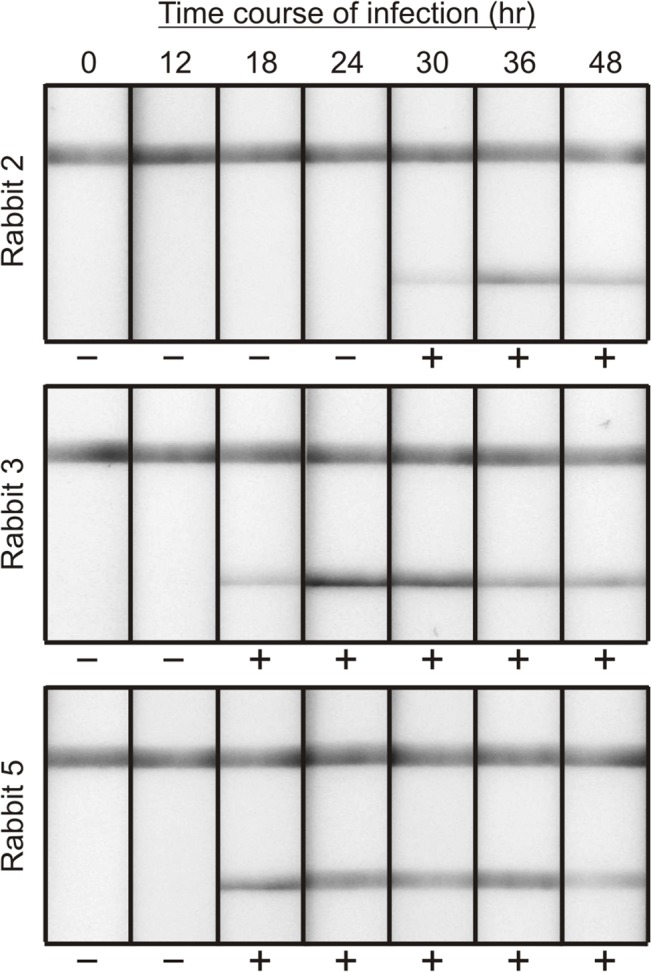
Use of LFA for detection of PGA in plasma throughout the time course of infection in three representative rabbits. The top line is a control indicating proper flow of the lateral flow device. The bottom line is the test line for detection of PGA. The indicated time is the number of hours after inhalational infection when blood was collected. Results of visual evaluation by four individuals are reported as negative (-) or positive (+) below each assay.

With an established sensitivity well below that needed to diagnose infection at or before a positive blood culture, the rapid anthrax diagnostic assay was then used to test plasma from infected rabbits that had been previously analyzed by the quantitative ELISA (from Fig 1). LFA done with sequential plasma samples from three representative rabbits are shown as an example of early PGA detection (Fig 3). The anthrax LFA results were positive for all rabbits at the point when *B*. *anthracis* was identified in blood cultures; importantly, the LFA was positive in 6 of the 10 rabbits before cultures showed the presence of bacteremia (*P* = 0.002, culture vs. LFA by One Way Repeated Measures ANOVA with post hoc evaluation by Bonferroni *t*-test) (Table 1).

The proven effectiveness of *B*. *anthracis* as a bioweapon and the narrow window for treatment of infection underscores the need for a test that can provide an earlier diagnosis than the current method of blood culture. The paucity of clinical samples dictates a reliance on animal models that are validated surrogates for studying *B*. *anthracis* disease in humans. In this rabbit model of inhalational anthrax, the anthrax LFA demonstrated the ability to detect PGA no later than, and up to, 12 hours before parallel blood samples produced positive cultures. Further, taking into account the time required for each diagnostic to yield definitive results (rapid anthrax diagnostic with immediate results vs. blood culture that requires at least 24 hours to confirm no growth), the anthrax LFA was capable of providing a diagnosis as many as 24 to 36 hours before culture in this model. Use of the PGA LFA with a somewhat increased sensitivity and a considerably decreased time to result compared to blood culture could greatly increase the chances for successful antibiotic therapy before the disease progresses beyond the point where treatment can alter the course of disease.

The anthrax bioterrorism attack in 2001 also identified the need for a rapid point-of-care diagnostic when clinical laboratories were overwhelmed by the influx of samples [13]. The anthrax LFA would allow for an on-site diagnosis of active infection, reducing the burden on healthcare resources and allowing medical providers to quickly identify patients who require immediate therapy. Indeed, with the 2012 FDA clearance of Raxibacumab, a monoclonal antibody that targets protective antigen for treatment of inhalational anthrax, screening of patients for the presence of PGA could identify patients in greatest need of a potentially limited therapeutic in the case of a mass exposure incident [14]. Finally, the simplicity of the assay’s design makes it capable of being utilized outside a hospital or laboratory setting, as a mass exposure event may necessitate.

In summary, immunoassay showed that PGA was a reliable biomarker for early diagnosis of inhalational anthrax in a rabbit model of disease. The low cost, rapid time to result, and ease of use make the PGA LFA well suited for responding to a mass exposure event. When used with individuals with potential exposure, the PGA LFA could provide early diagnosis for an infection that typically presents with flu-like symptoms, quickly identifying those most in need of treatment and greatly improving patient outcome.
